# Tetra­butyl­ammonium tris­(methyl­sulfanylmeth­yl)phenyl­borate

**DOI:** 10.1107/S1600536809054026

**Published:** 2009-12-19

**Authors:** Jan Tillmann, Hans-Wolfram Lerner, Michael Bolte

**Affiliations:** a Institut für Anorganische Chemie der Goethe-Universität Frankfurt, Max-von-Laue-Strasse 7, D-60438 Frankfurt am Main, Germany

## Abstract

In the title molecular salt, C_16_H_36_N^+^·C_12_H_20_BS_3_^−^, three of the four *n*-butyl chains show a *trans* conformation, whereas the fourth has the C—C—C—C torsion angle in a *gauche* conformation [−77.8 (5)°]. In the crystal, mol­ecules are packed in layers parallel to the (101) plane.

## Related literature

For the synthesis and properties of complexes with [(methyl­thio)meth­yl]borate ligands, see: Ohrenberg *et al.* (1996 ▶); Ruth *et al.* (2008 ▶).
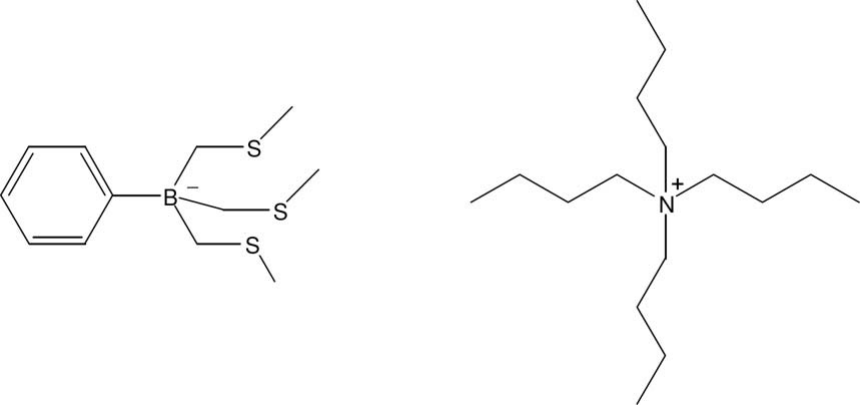


## Experimental

### 

#### Crystal data

C_16_H_36_N^+^·C_12_H_20_BS_3_^−^
*M* *_r_* = 513.73
Monoclinic, 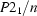

*a* = 9.8449 (8) Å
*b* = 15.6802 (9) Å
*c* = 20.8870 (17) Å
β = 92.215 (7)°
*V* = 3221.9 (4) Å^3^
*Z* = 4
Mo *K*α radiation
μ = 0.25 mm^−1^
*T* = 173 K
0.42 × 0.39 × 0.38 mm

#### Data collection

Stoe IPDS-II two-circle diffractometer
Absorption correction: multi-scan (*MULABS*; Spek, 2003; Blessing, 1995 ▶) *T*_min_ = 0.904, *T*_max_ = 0.912
17239 measured reflections
5999 independent reflections
4342 reflections with *I* > 2σ(*I*)
*R*_int_ = 0.066

#### Refinement

*R*[*F* ^2^ > 2σ(*F* ^2^)] = 0.055
*wR*(*F* ^2^) = 0.155
*S* = 1.01
5999 reflections
298 parameters
H-atom parameters constrained
Δρ_max_ = 0.72 e Å^−3^
Δρ_min_ = −0.32 e Å^−3^

### 

Data collection: *X-AREA* (Stoe & Cie, 2001 ▶); cell refinement: *X-AREA*; data reduction: *X-AREA*; program(s) used to solve structure: *SHELXS97* (Sheldrick, 2008 ▶); program(s) used to refine structure: *SHELXL97* (Sheldrick, 2008 ▶); molecular graphics: *XP* (Sheldrick, 2008 ▶); software used to prepare material for publication: *SHELXL97*.

## Supplementary Material

Crystal structure: contains datablocks I, global. DOI: 10.1107/S1600536809054026/vm2016sup1.cif

Structure factors: contains datablocks I. DOI: 10.1107/S1600536809054026/vm2016Isup2.hkl

Additional supplementary materials:  crystallographic information; 3D view; checkCIF report

## Figures and Tables

**Fig. 1.**
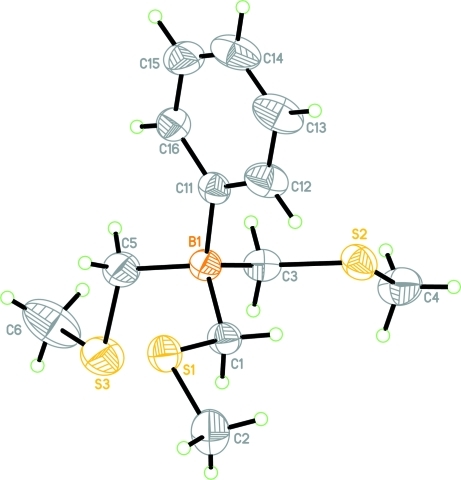
A view of the anion of the title compound, with the atom-numbering scheme. Displacement ellipsoids are drawn at the 50% probability level and H atoms are shown as small spheres of arbitrary radii.

**Fig. 2.**
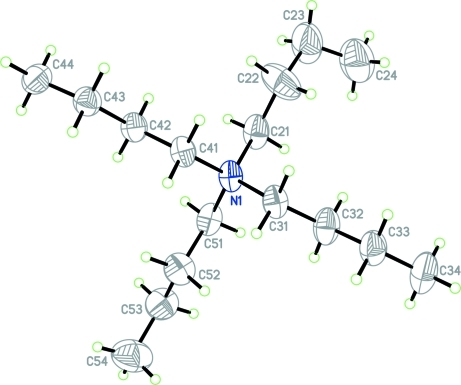
A view of the cation of the title compound, with the atom-numbering scheme. Displacement ellipsoids are drawn at the 50% probability level and H atoms are shown as small spheres of arbitrary radii.

**Fig. 3.**
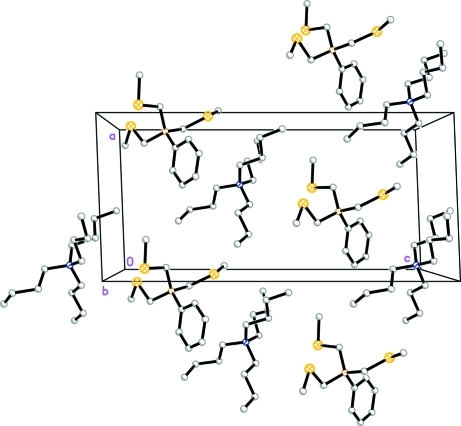
Crystal packing of the title compound with view along the b axis. H atoms are omitted for clarity.

**Fig. 4.**
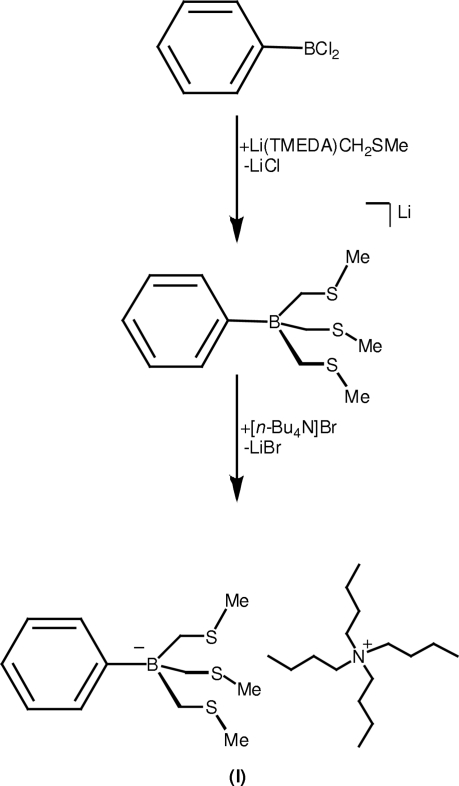
The formation of the title compound.

## supplementary crystallographic information

### Comment

Recently we have described the synthesis and properties of complexes with [(methylthio)methyl]borate ligands (C_6_H_5_)Bpz*_x_*(CH_2_SMe)_3-_*_x_* (Ruth *et al.*, 2008). It is interesting to note that these ligands facilitate an evaluation of the influence of gradual changes in the donor set of tripods on the chemical properties of the coordinated metal ion. Herein we report the crystal structure of the tetrabutylamonium [tris(methylthio)methyl]phenylborate [*n*-Bu_4_N][(C_6_H_5_)B(CH_2_SMe)_3_] (I). According to a literature procedure (Ohrenberg *et al.*, 1996), the tetrabutylamonium [tris(methylthio)methyl]phenylborate [*n*-Bu_4_N][(C_6_H_5_)B(CH_2_SMe)_3_] was easily accessible from the reaction of PhBCl_2_ with Li(TMEDA)CH_2_SMe (TMEDA: N,N,N',N'-tetramethylethylenediamine) and a subsequent cation exchange, as shown in equation below.

The title compound (Figs. 1 and 2) features discrete cations and anions. Three of the four *n*-butyl chains show an all *trans* conformation whereas the forth has one torsion angle in a *gauche* conformation. In the crystal the molecules are packed in layers parallel to the (1 0 1) plane (Figure 3).

### Experimental

(CH_3_)_2_S (19 mL, 259 mmol) and TMEDA (24 mL,160 mmol) were combined under a N_2_ atmosphere. n-BuLi (60 mL, 1.6 M in hexane) was added dropwise at -78°C. After 1 h at 25°C, the solution was heated at 45°C for 30 min to drive off unreacted (CH_3_)_2_S. The solution was again cooled to -78°C and (C_6_H_5_)BCl_2_ (3.3 mL, 25 mmol) was added dropwise. The mixture was allowed to warm to 25°C and was then stirred for 48 h. After the reaction was terminated all volatiles were removed in vacuo and the residue was treated with 200 mL of H_2_O and 70 mL of CH_2_Cl_2_. The aqueous solution was filtered, and the product (I) was precipitated by addition of aqueous [n-Bu_4_N]Br. The flocculent white product was isolated by filtration, washed with Et_2_O (2 x 10 mL), and dried under vacuum. Single crystals were obtained by recrystallisation from Et_2_O. ^1^H NMR (CDCl_3_): δ 8.09 (d, CH, 2 H), 7.27 (t, CH, 2 H), 7.05 (t, CH, 1 H), 2.57 -2.48 (m, BCH_2_ / NCH_2_, 14H ), 2.28 (s, SCH_3_, 9 H), 1.20 -1.17 (m, NCH_2_CH_2_CH_2_CH_3_, 16 H), 1.35 (m, CH_2_, 16 H), 0.87 (t, CH3, 12 H). ^11^B{^1^H} NMR (CDCl_3_): δ -14.4. Yield: 8.0 g (63%).

### Refinement

Hydrogen atoms were located in a difference Fourier map but they were included in calculated positions [C—H = 0.95 - 0.99 Å] and refined as riding [*U*_iso_(H) = 1.2*U*_eq_(C) or *U*_iso_(H) = 1.5*U*_eq_(C_methyl_)].

### Crystal data

**Table:**

C_16_H_36_N^+^·C_12_H_20_BS_3_^−^	*F*(000) = 1136
*M**_r_* = 513.73	*D*_x_ = 1.059 Mg m^−^^3^
Monoclinic, *P*2_1_/*n*	Mo *K*α radiation, λ = 0.71073 Å
Hall symbol: -P 2yn	Cell parameters from 13831 reflections
*a* = 9.8449 (8) Å	θ = 3.5–25.9°
*b* = 15.6802 (9) Å	µ = 0.25 mm^−^^1^
*c* = 20.8870 (17) Å	*T* = 173 K
β = 92.215 (7)°	Block, colourless
*V* = 3221.9 (4) Å^3^	0.42 × 0.39 × 0.38 mm
*Z* = 4	

### Data collection

**Table:**

Stoe IPDS-II two-circle diffractometer	5999 independent reflections
Radiation source: fine-focus sealed tube	4342 reflections with *I* > 2σ(*I*)
graphite	*R*_int_ = 0.066
ω scans	θ_max_ = 25.8°, θ_min_ = 3.4°
Absorption correction: multi-scan (*MULABS*; Spek, 2003; Blessing, 1995)	*h* = −10→11
*T*_min_ = 0.904, *T*_max_ = 0.912	*k* = −16→19
17239 measured reflections	*l* = −25→25

### Refinement

**Table:**

Refinement on *F*^2^	Primary atom site location: structure-invariant direct methods
Least-squares matrix: full	Secondary atom site location: difference Fourier map
*R*[*F*^2^ > 2σ(*F*^2^)] = 0.055	Hydrogen site location: inferred from neighbouring sites
*wR*(*F*^2^) = 0.155	H-atom parameters constrained
*S* = 1.01	*w* = 1/[σ^2^(*F*_o_^2^) + (0.0983*P*)^2^] where *P* = (*F*_o_^2^ + 2*F*_c_^2^)/3
5999 reflections	(Δ/σ)_max_ = 0.001
298 parameters	Δρ_max_ = 0.72 e Å^−^^3^
0 restraints	Δρ_min_ = −0.32 e Å^−^^3^

### Special details

**Table:**

Geometry. All e.s.d.'s (except the e.s.d. in the dihedral angle between two l.s. planes) are estimated using the full covariance matrix. The cell e.s.d.'s are taken into account individually in the estimation of e.s.d.'s in distances, angles and torsion angles; correlations between e.s.d.'s in cell parameters are only used when they are defined by crystal symmetry. An approximate (isotropic) treatment of cell e.s.d.'s is used for estimating e.s.d.'s involving l.s. planes.
Refinement. Refinement of *F*^2^ against ALL reflections. The weighted *R*-factor *wR* and goodness of fit *S* are based on *F*^2^, conventional *R*-factors *R* are based on *F*, with *F* set to zero for negative *F*^2^. The threshold expression of *F*^2^ > σ(*F*^2^) is used only for calculating *R*-factors(gt) *etc*. and is not relevant to the choice of reflections for refinement. *R*-factors based on *F*^2^ are statistically about twice as large as those based on *F*, and *R*- factors based on ALL data will be even larger.

### Fractional atomic coordinates and isotropic or equivalent isotropic displacement parameters (Å^2^)

**Table:**

	*x*	*y*	*z*	*U*_iso_*/*U*_eq_	
B1	0.4152 (3)	0.77978 (17)	0.68202 (11)	0.0359 (5)	
S1	0.56606 (7)	0.65306 (4)	0.61210 (3)	0.04352 (18)	
S2	0.51768 (7)	0.83143 (4)	0.80904 (3)	0.04459 (19)	
S3	0.46045 (9)	0.89280 (6)	0.57424 (4)	0.0682 (3)	
C1	0.5653 (2)	0.73852 (16)	0.66989 (10)	0.0383 (5)	
H1A	0.6039	0.7169	0.7112	0.046*	
H1B	0.6260	0.7844	0.6554	0.046*	
C2	0.7422 (3)	0.6237 (2)	0.61991 (13)	0.0580 (7)	
H2A	0.7602	0.5766	0.5906	0.087*	
H2B	0.7639	0.6059	0.6641	0.087*	
H2C	0.7988	0.6728	0.6093	0.087*	
C3	0.4401 (3)	0.86025 (15)	0.73206 (11)	0.0397 (5)	
H3A	0.3514	0.8877	0.7392	0.048*	
H3B	0.4986	0.9029	0.7116	0.048*	
C4	0.5650 (3)	0.9351 (2)	0.83934 (14)	0.0584 (7)	
H4A	0.6092	0.9291	0.8820	0.088*	
H4B	0.4835	0.9706	0.8423	0.088*	
H4C	0.6280	0.9620	0.8103	0.088*	
C5	0.3466 (3)	0.8213 (2)	0.61550 (12)	0.0547 (7)	
H5A	0.2636	0.8532	0.6262	0.066*	
H5B	0.3188	0.7744	0.5861	0.066*	
C6	0.3446 (5)	0.9760 (2)	0.54940 (17)	0.0845 (12)	
H6A	0.3936	1.0197	0.5260	0.127*	
H6B	0.3050	1.0016	0.5872	0.127*	
H6C	0.2721	0.9520	0.5215	0.127*	
C11	0.3093 (2)	0.71146 (14)	0.71190 (9)	0.0348 (5)	
C12	0.3530 (3)	0.64613 (18)	0.75270 (13)	0.0515 (6)	
H12	0.4479	0.6392	0.7611	0.062*	
C13	0.2638 (4)	0.59029 (19)	0.78188 (15)	0.0620 (8)	
H13	0.2981	0.5471	0.8100	0.074*	
C14	0.1245 (3)	0.59827 (18)	0.76956 (14)	0.0593 (8)	
H14	0.0629	0.5601	0.7886	0.071*	
C15	0.0777 (3)	0.6619 (2)	0.72964 (13)	0.0573 (7)	
H15	−0.0173	0.6683	0.7212	0.069*	
C16	0.1677 (3)	0.71714 (18)	0.70135 (11)	0.0449 (6)	
H16	0.1322	0.7606	0.6737	0.054*	
N1	0.5861 (2)	0.65794 (14)	0.39600 (8)	0.0396 (5)	
C21	0.5628 (3)	0.6327 (2)	0.32574 (10)	0.0531 (7)	
H21A	0.5438	0.6850	0.3005	0.064*	
H21B	0.6480	0.6078	0.3104	0.064*	
C22	0.4488 (4)	0.5700 (3)	0.31219 (14)	0.0858 (12)	
H22A	0.3639	0.5933	0.3291	0.103*	
H22B	0.4698	0.5161	0.3352	0.103*	
C23	0.4255 (5)	0.5507 (3)	0.24087 (17)	0.1044 (16)	
H23A	0.5147	0.5425	0.2215	0.125*	
H23B	0.3743	0.4966	0.2361	0.125*	
C24	0.3540 (5)	0.6152 (3)	0.20706 (19)	0.1017 (14)	
H24A	0.3438	0.5994	0.1617	0.153*	
H24B	0.4045	0.6690	0.2111	0.153*	
H24C	0.2640	0.6223	0.2248	0.153*	
C31	0.4585 (2)	0.69867 (18)	0.42244 (10)	0.0432 (6)	
H31A	0.3856	0.6552	0.4219	0.052*	
H31B	0.4785	0.7144	0.4677	0.052*	
C32	0.4051 (3)	0.7766 (2)	0.38756 (12)	0.0551 (7)	
H32A	0.3894	0.7630	0.3416	0.066*	
H32B	0.4734	0.8228	0.3911	0.066*	
C33	0.2732 (3)	0.8065 (2)	0.41532 (13)	0.0550 (7)	
H33A	0.2051	0.7602	0.4115	0.066*	
H33B	0.2891	0.8192	0.4614	0.066*	
C34	0.2171 (4)	0.8853 (3)	0.38171 (17)	0.0761 (10)	
H34A	0.1329	0.9029	0.4015	0.114*	
H34B	0.1982	0.8725	0.3363	0.114*	
H34C	0.2839	0.9315	0.3856	0.114*	
C41	0.6161 (2)	0.58012 (17)	0.43699 (10)	0.0404 (5)	
H41A	0.5374	0.5410	0.4325	0.048*	
H41B	0.6234	0.5985	0.4823	0.048*	
C42	0.7434 (3)	0.53021 (17)	0.42250 (11)	0.0441 (6)	
H42A	0.8242	0.5673	0.4287	0.053*	
H42B	0.7385	0.5110	0.3773	0.053*	
C43	0.7567 (3)	0.45311 (17)	0.46679 (11)	0.0464 (6)	
H43A	0.7564	0.4729	0.5118	0.056*	
H43B	0.6767	0.4157	0.4593	0.056*	
C44	0.8850 (3)	0.40157 (19)	0.45726 (13)	0.0570 (7)	
H44A	0.8882	0.3533	0.4871	0.086*	
H44B	0.9648	0.4379	0.4654	0.086*	
H44C	0.8848	0.3802	0.4132	0.086*	
C51	0.7038 (3)	0.72033 (18)	0.39848 (11)	0.0454 (6)	
H51A	0.6781	0.7702	0.3716	0.054*	
H51B	0.7824	0.6927	0.3788	0.054*	
C52	0.7496 (3)	0.7524 (2)	0.46443 (13)	0.0550 (7)	
H52A	0.6735	0.7825	0.4843	0.066*	
H52B	0.7762	0.7035	0.4921	0.066*	
C53	0.8687 (3)	0.8124 (2)	0.45910 (16)	0.0638 (8)	
H53A	0.8417	0.8602	0.4304	0.077*	
H53B	0.9442	0.7816	0.4394	0.077*	
C54	0.9186 (5)	0.8482 (3)	0.5233 (2)	0.0880 (12)	
H54A	0.9946	0.8873	0.5169	0.132*	
H54B	0.9490	0.8014	0.5515	0.132*	
H54C	0.8445	0.8791	0.5431	0.132*	

### Atomic displacement parameters (Å^2^)

**Table:**

	*U*^11^	*U*^22^	*U*^33^	*U*^12^	*U*^13^	*U*^23^
B1	0.0354 (13)	0.0366 (14)	0.0357 (11)	−0.0025 (12)	0.0024 (10)	0.0031 (10)
S1	0.0477 (4)	0.0445 (4)	0.0385 (3)	−0.0020 (3)	0.0025 (2)	−0.0098 (2)
S2	0.0522 (4)	0.0429 (4)	0.0391 (3)	−0.0079 (3)	0.0075 (2)	−0.0054 (2)
S3	0.0601 (5)	0.0804 (6)	0.0645 (4)	−0.0095 (4)	0.0053 (3)	0.0372 (4)
C1	0.0387 (13)	0.0389 (13)	0.0375 (10)	−0.0030 (11)	0.0055 (9)	−0.0081 (9)
C2	0.0628 (19)	0.0598 (18)	0.0513 (14)	0.0236 (15)	−0.0014 (12)	−0.0098 (12)
C3	0.0382 (13)	0.0340 (12)	0.0474 (12)	0.0010 (10)	0.0069 (10)	0.0019 (9)
C4	0.0533 (17)	0.0574 (18)	0.0649 (16)	−0.0126 (14)	0.0093 (13)	−0.0234 (13)
C5	0.0454 (15)	0.0674 (19)	0.0512 (14)	−0.0094 (14)	−0.0001 (11)	0.0208 (13)
C6	0.117 (3)	0.057 (2)	0.082 (2)	0.017 (2)	0.044 (2)	0.0157 (17)
C11	0.0393 (12)	0.0332 (12)	0.0320 (10)	−0.0026 (10)	0.0050 (8)	−0.0060 (8)
C12	0.0507 (16)	0.0463 (15)	0.0587 (14)	0.0045 (13)	0.0155 (12)	0.0108 (12)
C13	0.079 (2)	0.0410 (15)	0.0687 (17)	0.0040 (15)	0.0302 (15)	0.0095 (13)
C14	0.073 (2)	0.0422 (15)	0.0654 (16)	−0.0237 (15)	0.0324 (15)	−0.0152 (13)
C15	0.0453 (15)	0.071 (2)	0.0565 (15)	−0.0218 (15)	0.0073 (12)	−0.0138 (14)
C16	0.0412 (14)	0.0538 (15)	0.0399 (11)	−0.0074 (12)	0.0029 (10)	−0.0045 (10)
N1	0.0335 (10)	0.0543 (13)	0.0313 (9)	0.0039 (9)	0.0056 (7)	0.0103 (8)
C21	0.0553 (16)	0.0746 (19)	0.0297 (11)	0.0172 (15)	0.0045 (10)	0.0087 (11)
C22	0.098 (3)	0.110 (3)	0.0477 (16)	−0.026 (3)	−0.0208 (16)	−0.0047 (17)
C23	0.108 (3)	0.134 (4)	0.068 (2)	0.052 (3)	−0.033 (2)	−0.039 (2)
C24	0.099 (3)	0.130 (4)	0.075 (2)	0.010 (3)	−0.016 (2)	0.010 (2)
C31	0.0317 (12)	0.0621 (16)	0.0362 (11)	0.0033 (12)	0.0072 (9)	0.0077 (10)
C32	0.0435 (15)	0.073 (2)	0.0490 (13)	0.0127 (14)	0.0092 (11)	0.0139 (13)
C33	0.0408 (15)	0.0704 (19)	0.0537 (14)	0.0105 (14)	0.0023 (11)	0.0007 (13)
C34	0.059 (2)	0.088 (3)	0.082 (2)	0.0294 (19)	0.0013 (16)	0.0093 (18)
C41	0.0369 (13)	0.0511 (14)	0.0333 (10)	−0.0015 (11)	0.0025 (9)	0.0089 (9)
C42	0.0432 (14)	0.0498 (15)	0.0395 (11)	0.0011 (12)	0.0024 (10)	0.0050 (10)
C43	0.0500 (15)	0.0467 (15)	0.0421 (12)	−0.0023 (12)	−0.0018 (10)	0.0039 (10)
C44	0.069 (2)	0.0479 (16)	0.0543 (14)	0.0092 (15)	−0.0008 (13)	−0.0012 (12)
C51	0.0364 (13)	0.0525 (15)	0.0481 (12)	0.0035 (12)	0.0133 (10)	0.0163 (11)
C52	0.0457 (16)	0.0625 (18)	0.0573 (15)	−0.0082 (14)	0.0094 (12)	0.0043 (13)
C53	0.0512 (18)	0.0590 (19)	0.082 (2)	−0.0114 (15)	0.0116 (15)	0.0012 (15)
C54	0.082 (3)	0.081 (3)	0.101 (3)	−0.024 (2)	0.003 (2)	−0.019 (2)

### Geometric parameters (Å, °)

**Table:**

B1—C11	1.635 (3)	C22—H22A	0.9900
B1—C1	1.642 (4)	C22—H22B	0.9900
B1—C3	1.651 (3)	C23—C24	1.406 (6)
B1—C5	1.655 (3)	C23—H23A	0.9900
S1—C2	1.796 (3)	C23—H23B	0.9900
S1—C1	1.804 (2)	C24—H24A	0.9800
S2—C4	1.799 (3)	C24—H24B	0.9800
S2—C3	1.811 (2)	C24—H24C	0.9800
S3—C6	1.796 (4)	C31—C32	1.507 (4)
S3—C5	1.825 (3)	C31—H31A	0.9900
C1—H1A	0.9900	C31—H31B	0.9900
C1—H1B	0.9900	C32—C33	1.517 (4)
C2—H2A	0.9800	C32—H32A	0.9900
C2—H2B	0.9800	C32—H32B	0.9900
C2—H2C	0.9800	C33—C34	1.514 (4)
C3—H3A	0.9900	C33—H33A	0.9900
C3—H3B	0.9900	C33—H33B	0.9900
C4—H4A	0.9800	C34—H34A	0.9800
C4—H4B	0.9800	C34—H34B	0.9800
C4—H4C	0.9800	C34—H34C	0.9800
C5—H5A	0.9900	C41—C42	1.518 (4)
C5—H5B	0.9900	C41—H41A	0.9900
C6—H6A	0.9800	C41—H41B	0.9900
C6—H6B	0.9800	C42—C43	1.525 (3)
C6—H6C	0.9800	C42—H42A	0.9900
C11—C12	1.390 (4)	C42—H42B	0.9900
C11—C16	1.406 (3)	C43—C44	1.519 (4)
C12—C13	1.396 (4)	C43—H43A	0.9900
C12—H12	0.9500	C43—H43B	0.9900
C13—C14	1.391 (5)	C44—H44A	0.9800
C13—H13	0.9500	C44—H44B	0.9800
C14—C15	1.369 (5)	C44—H44C	0.9800
C14—H14	0.9500	C51—C52	1.518 (4)
C15—C16	1.387 (4)	C51—H51A	0.9900
C15—H15	0.9500	C51—H51B	0.9900
C16—H16	0.9500	C52—C53	1.510 (4)
N1—C41	1.513 (3)	C52—H52A	0.9900
N1—C51	1.516 (3)	C52—H52B	0.9900
N1—C21	1.529 (3)	C53—C54	1.519 (5)
N1—C31	1.531 (3)	C53—H53A	0.9900
C21—C22	1.511 (5)	C53—H53B	0.9900
C21—H21A	0.9900	C54—H54A	0.9800
C21—H21B	0.9900	C54—H54B	0.9800
C22—C23	1.529 (4)	C54—H54C	0.9800
			
C11—B1—C1	113.1 (2)	C22—C23—H23A	108.8
C11—B1—C3	109.90 (17)	C24—C23—H23B	108.8
C1—B1—C3	106.64 (19)	C22—C23—H23B	108.8
C11—B1—C5	109.4 (2)	H23A—C23—H23B	107.7
C1—B1—C5	111.48 (19)	C23—C24—H24A	109.5
C3—B1—C5	106.1 (2)	C23—C24—H24B	109.5
C2—S1—C1	99.11 (13)	H24A—C24—H24B	109.5
C4—S2—C3	100.46 (13)	C23—C24—H24C	109.5
C6—S3—C5	100.82 (16)	H24A—C24—H24C	109.5
B1—C1—S1	115.02 (16)	H24B—C24—H24C	109.5
B1—C1—H1A	108.5	C32—C31—N1	116.09 (18)
S1—C1—H1A	108.5	C32—C31—H31A	108.3
B1—C1—H1B	108.5	N1—C31—H31A	108.3
S1—C1—H1B	108.5	C32—C31—H31B	108.3
H1A—C1—H1B	107.5	N1—C31—H31B	108.3
S1—C2—H2A	109.5	H31A—C31—H31B	107.4
S1—C2—H2B	109.5	C31—C32—C33	110.8 (2)
H2A—C2—H2B	109.5	C31—C32—H32A	109.5
S1—C2—H2C	109.5	C33—C32—H32A	109.5
H2A—C2—H2C	109.5	C31—C32—H32B	109.5
H2B—C2—H2C	109.5	C33—C32—H32B	109.5
B1—C3—S2	114.75 (16)	H32A—C32—H32B	108.1
B1—C3—H3A	108.6	C34—C33—C32	112.2 (2)
S2—C3—H3A	108.6	C34—C33—H33A	109.2
B1—C3—H3B	108.6	C32—C33—H33A	109.2
S2—C3—H3B	108.6	C34—C33—H33B	109.2
H3A—C3—H3B	107.6	C32—C33—H33B	109.2
S2—C4—H4A	109.5	H33A—C33—H33B	107.9
S2—C4—H4B	109.5	C33—C34—H34A	109.5
H4A—C4—H4B	109.5	C33—C34—H34B	109.5
S2—C4—H4C	109.5	H34A—C34—H34B	109.5
H4A—C4—H4C	109.5	C33—C34—H34C	109.5
H4B—C4—H4C	109.5	H34A—C34—H34C	109.5
B1—C5—S3	113.57 (18)	H34B—C34—H34C	109.5
B1—C5—H5A	108.9	N1—C41—C42	116.60 (18)
S3—C5—H5A	108.9	N1—C41—H41A	108.1
B1—C5—H5B	108.9	C42—C41—H41A	108.1
S3—C5—H5B	108.9	N1—C41—H41B	108.1
H5A—C5—H5B	107.7	C42—C41—H41B	108.1
S3—C6—H6A	109.5	H41A—C41—H41B	107.3
S3—C6—H6B	109.5	C41—C42—C43	109.9 (2)
H6A—C6—H6B	109.5	C41—C42—H42A	109.7
S3—C6—H6C	109.5	C43—C42—H42A	109.7
H6A—C6—H6C	109.5	C41—C42—H42B	109.7
H6B—C6—H6C	109.5	C43—C42—H42B	109.7
C12—C11—C16	115.1 (2)	H42A—C42—H42B	108.2
C12—C11—B1	122.0 (2)	C44—C43—C42	113.2 (2)
C16—C11—B1	122.8 (2)	C44—C43—H43A	108.9
C11—C12—C13	123.0 (3)	C42—C43—H43A	108.9
C11—C12—H12	118.5	C44—C43—H43B	108.9
C13—C12—H12	118.5	C42—C43—H43B	108.9
C14—C13—C12	119.6 (3)	H43A—C43—H43B	107.7
C14—C13—H13	120.2	C43—C44—H44A	109.5
C12—C13—H13	120.2	C43—C44—H44B	109.5
C15—C14—C13	119.0 (3)	H44A—C44—H44B	109.5
C15—C14—H14	120.5	C43—C44—H44C	109.5
C13—C14—H14	120.5	H44A—C44—H44C	109.5
C14—C15—C16	120.6 (3)	H44B—C44—H44C	109.5
C14—C15—H15	119.7	N1—C51—C52	116.39 (18)
C16—C15—H15	119.7	N1—C51—H51A	108.2
C15—C16—C11	122.6 (3)	C52—C51—H51A	108.2
C15—C16—H16	118.7	N1—C51—H51B	108.2
C11—C16—H16	118.7	C52—C51—H51B	108.2
C41—N1—C51	111.63 (18)	H51A—C51—H51B	107.3
C41—N1—C21	110.7 (2)	C53—C52—C51	110.1 (2)
C51—N1—C21	106.63 (18)	C53—C52—H52A	109.6
C41—N1—C31	106.10 (16)	C51—C52—H52A	109.6
C51—N1—C31	110.8 (2)	C53—C52—H52B	109.6
C21—N1—C31	111.09 (18)	C51—C52—H52B	109.6
C22—C21—N1	115.5 (2)	H52A—C52—H52B	108.2
C22—C21—H21A	108.4	C52—C53—C54	113.0 (3)
N1—C21—H21A	108.4	C52—C53—H53A	109.0
C22—C21—H21B	108.4	C54—C53—H53A	109.0
N1—C21—H21B	108.4	C52—C53—H53B	109.0
H21A—C21—H21B	107.5	C54—C53—H53B	109.0
C21—C22—C23	113.2 (3)	H53A—C53—H53B	107.8
C21—C22—H22A	108.9	C53—C54—H54A	109.5
C23—C22—H22A	108.9	C53—C54—H54B	109.5
C21—C22—H22B	108.9	H54A—C54—H54B	109.5
C23—C22—H22B	108.9	C53—C54—H54C	109.5
H22A—C22—H22B	107.8	H54A—C54—H54C	109.5
C24—C23—C22	113.6 (4)	H54B—C54—H54C	109.5
C24—C23—H23A	108.8		
			
C11—B1—C1—S1	63.1 (2)	C14—C15—C16—C11	−0.2 (4)
C3—B1—C1—S1	−176.06 (15)	C12—C11—C16—C15	0.2 (3)
C5—B1—C1—S1	−60.7 (3)	B1—C11—C16—C15	−176.3 (2)
C2—S1—C1—B1	−175.47 (18)	C41—N1—C21—C22	58.3 (3)
C11—B1—C3—S2	62.7 (2)	C51—N1—C21—C22	179.9 (3)
C1—B1—C3—S2	−60.2 (2)	C31—N1—C21—C22	−59.3 (3)
C5—B1—C3—S2	−179.10 (16)	N1—C21—C22—C23	177.0 (3)
C4—S2—C3—B1	166.25 (18)	C21—C22—C23—C24	−77.8 (5)
C11—B1—C5—S3	−175.62 (18)	C41—N1—C31—C32	−177.9 (2)
C1—B1—C5—S3	−49.8 (3)	C51—N1—C31—C32	60.7 (3)
C3—B1—C5—S3	65.9 (2)	C21—N1—C31—C32	−57.6 (3)
C6—S3—C5—B1	−139.8 (2)	N1—C31—C32—C33	175.7 (2)
C1—B1—C11—C12	31.9 (3)	C31—C32—C33—C34	179.5 (3)
C3—B1—C11—C12	−87.1 (3)	C51—N1—C41—C42	−56.3 (3)
C5—B1—C11—C12	156.8 (2)	C21—N1—C41—C42	62.3 (3)
C1—B1—C11—C16	−151.8 (2)	C31—N1—C41—C42	−177.1 (2)
C3—B1—C11—C16	89.2 (3)	N1—C41—C42—C43	−178.7 (2)
C5—B1—C11—C16	−26.9 (3)	C41—C42—C43—C44	−177.6 (2)
C16—C11—C12—C13	−0.6 (4)	C41—N1—C51—C52	−56.9 (3)
B1—C11—C12—C13	176.0 (2)	C21—N1—C51—C52	−177.9 (2)
C11—C12—C13—C14	1.0 (4)	C31—N1—C51—C52	61.1 (3)
C12—C13—C14—C15	−1.0 (4)	N1—C51—C52—C53	178.9 (2)
C13—C14—C15—C16	0.6 (4)	C51—C52—C53—C54	179.1 (3)
